# CpG-Activated Regulatory B-Cell Progenitors Alleviate Murine Graft-Versus-Host-Disease

**DOI:** 10.3389/fimmu.2022.790564

**Published:** 2022-04-11

**Authors:** Viviane A. Agbogan, Pauline Gastineau, Emmanuel Tejerina, Saoussen Karray, Flora Zavala

**Affiliations:** ^1^Université Paris Cité, INSERM U1151, CNRS UMR8152, Institut Necker Enfants Malades (INEM), Paris, France; ^2^Université Paris Cité, INSERM U976, Institut de Recherche Saint-Louis (IRSL), Hôpital Saint-Louis, Paris, France

**Keywords:** allogeneic stem cell transplantation (allo-SCT), regulatory B-cell progenitors, CpG-proBs, cell therapy, fibrosis, Bregs: regulatory B cells, graft-versus host disease

## Abstract

Development of Graft Versus Host Disease (GVHD) represents a major impediment in allogeneic hematopoietic stem cell transplantation (HSCT). The observation that the presence of bone marrow and circulating hematogones correlated with reduced GVHD risks prompted us to evaluate whether B-cell progenitors, which provide protection in various autoimmune disease models following activation with the TLR-9 agonist CpG (CpG-proBs), could likewise reduce this allogeneic disorder. In a murine model of GVHD that recapitulates an initial phase of acute GVHD followed by a phase of chronic sclerodermatous GVHD, we found that CpG-proBs, adoptively transferred during the initial phase of disease, reduced the diarrhea score and mostly prevented cutaneous fibrosis. Progenitors migrated to the draining lymph nodes and to the skin where they mainly differentiated into follicular B cells. CpG activation and IFN-γ expression were required for the protective effect, which resulted in reduced CD4^+^ T-cell-derived production of critical cytokines such as TGF-β, IL-13 and IL-21. Adoptive transfer of CpG-proBs increased the T follicular regulatory to T follicular helper (Tfr/Tfh) ratio. Moreover, CpG-proBs privileged the accumulation of IL-10-positive CD8^+^ T cells, B cells and dendritic cells in the skin. However, CpG-proBs did not improve survival. Altogether, our findings support the notion that adoptively transferred CpG-proBs exert immunomodulating effect that alleviates symptoms of GVHD but require additional anti-inflammatory strategy to improve survival.

## Introduction

Graft-versus-host disease (GVHD), a donor cell-mediated immune disorder presenting in sequential acute and chronic forms, represents a major drawback for long-term effectiveness of allogeneic hematopoietic stem cell transplantation (HSCT) in hematologic malignancies. Efforts to improve immune regulation to prevent this disease have remained challenging. In addition to regulatory T cell deficiencies in both acute (1) and chronic (2, 3) GVHD, aberrant B cell homeostasis (4), with reduced generation of bone marrow (BM) B lymphoid progenitors (5), low frequencies of naive and memory cells, and a regulatory B cell (Breg) cell defect have recently been described (6, 7) in chronic GVHD. This led to the hypothesis that tolerogenic B-cell progenitors might play a role in the outcome of HSC transplantation. In accordance with this hypothesis, high numbers of donor BM B-cell progenitors were inversely correlated with the occurrence of GVHD in its acute (aGVHD) (8, 9) or chronic (cGVHD) form (10, 11) in HSC-transplanted patients. More recent studies have shown that their expansion at the time of engraftment heralded less frequent development of acute severe GVHD with increased mature B-cell counts and IgG levels post-HSCT (12, 13). Circulating B-cell progenitors have been detected in very low numbers in patients with low-grade acute GVHD scores (14). Whether they exhibit any suppressive properties either directly or by promoting the emergence of other regulatory cell types involved in GVHD inhibition remains unknown so far.

We have recently shown in mice that MyD88-dependent activation of BM cells by the Toll-like receptor-9 (TLR-9) agonist CpG-B as well as its injection *in vivo*, induced the emergence within the BM of a B-cell progenitor population, at the pro-B cell stage of differentiation, endowed with potent suppressive properties against autoreactive CD4^+^ T cells. Importantly, these progenitors migrated into the autoimmune reaction sites and differentiated *in vivo* into several more mature B-cell subsets, which also shared suppressive properties (15–17). This *in vivo* maturation of the CpG-proBs into suppressive Bregs may account for the long-lasting effect of a single injection of CpG-proBs as well as for their remarkable suppressive potency. Indeed, as few as 60,000 CpG-proBs injected once at the onset of clinical signs were able to provide protection against nonobese type 1 diabetes (T1D) (15) and EAE (16), a murine model of multiple sclerosis.

The efficacy of CpG-proBs in murine autoimmunity models prompted us to examine whether this activated population could likewise provide protection in an allogeneic setting, namely a murine model of GVHD (18) that has been reported as developing along sequential acute and chronic phases and also for sharing features of autoimmune inflammation. To this end, we evaluated the effect of CpG-proBs on GVHD in terms of severity of diarrhea, skin fibrosis and survival. We examined how these cells migrated into diverse sites of the allogeneic response, including mesenteric lymph nodes (mLN), peripheral lymph nodes (pLN) and skin and analyzed their differentiation into more mature B-cell subsets. We further assessed their capacity to modulate the cytokine profile during GVHD and determined which cytokines were required for protection. Finally, we investigated how the administration of CpG-proBs affected the T follicular regulatory (Tfr) to T follicular helper (Tfh) cell ratio (Tfr/Tfh), which is key in controlling the CD4^+^ T-B cell interaction taking place in GVHD.

## Materials and Methods

### Mice

Female Balb/c mice were obtained from Janvier Laboratories (Le Genest Saint Isle, France) and maintained under acidified water upon arrival. Donor cells were from specific pathogen free (SPF) C57BL/6J mice (from Janvier laboratories), congenic CD45.1^+^ C57BL/6J, Actin-GFP knock-in (KI) C57BL/6J, IFN-γ deficient C57BL/6J mice, all raised in our accredited animal facility at Institut Necker Enfants Malades under pathogen-free conditions. All mice were backcrossed for at least ten generations.

### GVHD Induction and Clinical Scoring

Balb/c mice (female, 10 wk-old) were irradiated at 5.8 Gy in a Faxitron X-Ray irradiator at day 0 and reconstituted at day+1 by i.v. retro-orbital injection with 5 x 10^6^ T- and B-cell-depleted BM cells as well as 1 x 10^6^ splenocytes from C57BL/6J donors. Clinical evolution of GVHD was scored over 60-80 days, for survival, diarrhea, weight, posture, mobility and skin damage (18).

### T- and B-Cell Depletion of BM Cells

Donor T- and B-cell-depleted (TBCD) BM cells were isolated by flushing femurs and tibias from donor mice with RPMI 1640. After centrifugation, cells were stained for 15 min with anti-CD3-PE and anti-CD19-PE in PBS, 2% FCS and rat anti-mouse IgG and sheep anti-mouse IgM were added. Depletion was completed with anti-rat and anti-sheep beads, respectively (ThermoFisher Scientific) after 3 passages over a magnet in 5ml tubes. The TBCD-BM fraction contains mainly myeloid, precursor and stem cells.

### B-Cell Progenitor Sorting and Expansion

CpG-proB cells were isolated from C57BL/6J BM cell cultures activated with 1 μM CpG-1668 (CpG-B) (Eurogentec, Angers, France) for 17h in low endotoxin-RPMI medium (Fisher Scientific, Illkirch, France) supplemented with 10% (vol/vol) FCS and 1% antibiotics (penicillin and streptomycin). c-kit^+^ cells were magnetically sorted using the Robosep automaton (StemCell Technologies, Grenoble, France) and thereafter stained with appropriately labeled mAbs and sorted by flow cytometry on a BD FACS Aria IIIu cell-sorter as c-kit^+^Sca-1^+^B220^+^PDCA-1^-^IgM^-^ cells. Electronically sorted B-cell progenitors were cultured on plates at 20,000 cells/ml over OP-9 stromal cells in OPTIMEM medium (Gibco) supplemented with 10% FCS, 1% antibiotics, 0.1% β-mercaptoethanol and 20 ng/ml Flt3L, SCF (Immunotools, Frisoythe, Germany) and IL-7 (Peprotech France, Neuilly-sur-Seine, France), achieving on average a 10-fold expansion of sorted CpG-proBs over 6 days. Expanded CpG-proBs were further stained and electronically sorted as c-kit^low/-^ Sca-1^+^B220^+^PDCA-1^-^IgM^-^ cells, routinely assessed as >95% pure, before i.v. injection through the retro-orbital sinus.

### Recovery of Cells From Lymph Nodes and Skin Samples

Inguinal (n=2), axillary and brachial (n=4), cervical (n=2) and mesenteric (n=3) lymph nodes were collected from GVHD controls and CpG-proB recipients, yielding equivalent cell counts in both groups. Skin samples were harvested and digested in RPMI medium (Fisher Scientific, Illkirch, France) supplemented with 1% (vol/vol) FCS, 1% antibiotics (penicillin and streptomycin), 1mg/mL collagenase D (Roche, Sigma COLLD-RO) and 1,000 IU DNase (Sigma-Aldrich, Fleury-Mérogis, France) for 45 min at 37°C [adapted from (19)].

### Flow Cytometry Analysis of Cell Subsets and Cytokine Expression

To block nonspecific Fc receptor binding, cells were pre-incubated for 10 min at room temperature with FcR blocker 2.4G2 mAb. Cells were then stained with appropriately labeled mAbs against CD4, B220, MHC II, PDCA-1, PDL-1, PDL-2, CD21, IgM, CD93, CD23, CD11b, F4/80 (eBioscience, ThermoFisher Scientific, Montigny-le-Bretonneux, France), c-Kit (CD117) (BioLegend, San Diego, CA), Sca-1 (anti-Ly6A/E), CD40, CD80, CD86, CD11c, CD8 (BD Bioscience/Pharmingen, Le Pont-de-Claix, France), CXCR5 (Sony, Weybridge, Surrey, UK) or GFP (ThermoFisher Scientific). Nuclear Foxp3 expression was measured by FACS analysis as per the manufacturer’s instructions (eBioscience, ThermoFisher Scientific). Positive cells were defined using an isotype control antibody. Intra-cytoplasmic cytokine expression was assessed after a 4-h stimulation with PMA (10 ng/ml) plus ionomycin (500 ng/ml) in the presence of Brefeldin A (2 mg/ml), followed by fixation/permeabilization with PFA/saponin and subsequent staining with specific antibodies including PE-labeled anti-TGF-β, PE-labeled anti-IL-27p28, PE-labeled anti-GM-CSF, APC-labeled anti-IL-10, APC-labeled anti–IFN-γ, APC-labelled anti-IL-21 (eBioscience), APC-labeled anti-IL-17 (BD bioscience), FITC-labeled anti-IL-6, PE-labeled anti-IL-13, APC-labeled anti-IL-4 (Sony) and FITC-labeled anti-TNF-α (Biolegend). Positive cells were defined using isotype Ab-stained controls (BD Biosciences and eBioscience). Membrane and intracellular antigen expression was analyzed in a FACS Canto II cytometer (BD Biosciences) using FlowJo software (Treestar, Ashland, OR).

### qRT-PCR Microarray Analysis in Skin Samples

Skin samples (2cm^2^) were collected from the back of GVHD controls or CpG-proB recipients at day+70, frozen in liquid nitrogen and stored at -80°C. Frozen tissues were then placed in Qiagen lysis buffer and dissociated using GentleMACS dissociator (Miltenyi Biotec, Paris, France). RNA was extracted with RNeasy Plus Universal mini-Kit (Qiagen, Courtaboeuf, France) following the manufacturer’s instructions. The A260/A280 values of all RNA samples ranged from 2.06-2.1. Production of cDNA from 1ng of total extracted RNA was performed using random primers (Invitrogen, ThermoFisher Scientific, Montigny-le Bretonneux, France) and reverse transcriptase superscript II (Life Technologies, Villebon-sur-Yvette, France). qRT-PCR array for measuring the expression of 80 genes of interest (and 8 house-keeping genes), targeting cytokines and fibrosis-related genes, was performed on a custom-made plate (Anygenes, Paris, France) with SYBRGreen, using a qTower2 thermal cycler (Analytic Jena, Jena, Germany). See **Supplementary Table 1** for information on primers used in the qRT-PCR array.

Analysis was performed with Qlucore software (Lund, Sweden). Results are expressed as 2-(delta delta Ct) and gene expression was normalized using the geometrical mean of 6 housekeeping genes. The threshold for the selection of differentially expressed genes was an expression fold-change ≥1.4 and a *p ≤* 0.05.

### Histology

Skin sections (4 μm thick) recovered from the back of mice at day+70 were fixed in 4% paraformaldehyde, embedded in paraffin and stained with H&E. Epidermal thickness was measured on scanned images with NDP.view software (Hamamatsu City, Japan).

### Statistics

Statistical analysis was performed using GraphPad Prism (GraphPad Software, La Jolla, CA). Normality and variance equality were assessed for every data set with Shapiro-Wilk test (for samples with n>5) or D’Agostino-Pearson (for samples with n ≤ 5) and F Test respectively. Survival curves were analyzed with Kaplan-Meier estimates. Disease curves and multiple cytokine production were analyzed using a two-way ANOVA test, with Bonferroni multiple comparison post-test. Cell proportions were analyzed using two-way ANOVA with Bonferroni multiple comparison, Student’s *t*-test or one-way ANOVA. Data are shown as mean ± SEM. *P* ≤ 0.05 was considered statistically significant.

## Results

### CpG-proBs Protect Against GVHD: Assessment of Cellular Dose and Therapeutic Window

**Figure 1:**
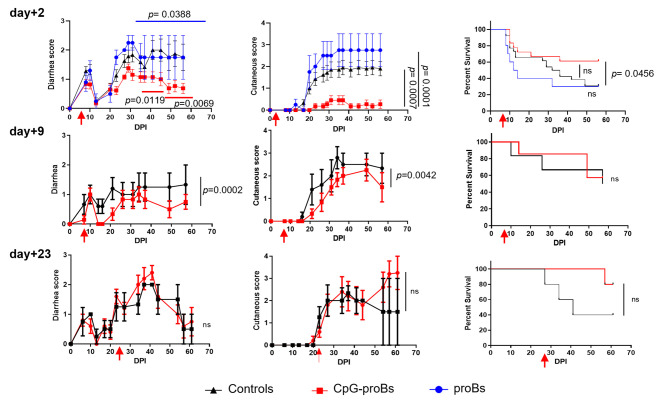
Effect of adoptively transferred CpG-proBs on GVHD symptoms. Balb/c recipients irradiated at 5.8 Gy on day-0, were reconstituted on day+1 with T- and B-cell depleted BM cells (5x10^6^ cells) and splenocytes (1 x 10^6^ cells) from C57BL/6J donors. CpG-proBs (7.5 x 10^5^cells) or proBs prepared from C57BL/6J donors and expanded in co-culture with OP-9 stromal cells were adoptively transferred on day+2, day+9 or day+23 post-irradiation (DPI) as indicated. Diarrhea, cutaneous scores and survival are shown over a period of 60-80 days. Results are expressed as means ± SEM. Adoptive transfer (or PBS injection in control GVHD mice) was performed on day+2 in GVHD control mice (N=30, black line), CpG-proB recipients (N=19, red line), proB recipients (N=10, blue line); on day+9, in GVHD controls (N=6, black line) and CpG-proB recipients (N=7, red line); on day+23, in GVHD controls (N=7, black line) and CpG-proB recipients (N=6, red line). Statistical analysis was performed with two-way ANOVA with Bonferroni post-tests for diarrhea score and cutaneous score and Kaplan-Meier estimates for survival; *p* values as indicated; ns=non significant.

After induction, GVHD went through an initial phase accompanied by diarrhea between day+2 and day+18 followed by a chronic stage from day+20 onwards, characterized by a second bout of diarrhea together with cutaneous manifestations. CpG-proBs were sorted as c-kit^+^Sca-1^+^B220^low^PDCA-1^-^IgM^-^ cells, as reported before (16) (**Supplementary Figure 1A**). A dose of 10^5^ CpG-proBs, previously shown to be effective in autoimmune settings, did not significantly reduce the severity of GVHD, when the adoptive transfer took place the day following reconstitution (**Supplementary Figure 1B**). To increase the amount of progenitors available for transfer, CpG-proBs were co-cultured with OP-9 stromal cells for 6 days. After a 10-fold expansion, on average, these progenitors were electronically sorted to routinely >95% purity. They shared a similar phenotype with CpG-proBs that had not been expanded, except for the loss of c-kit expression, presumably resulting from the presence of its ligand SCF in the expansion medium (**Supplementary Figure 1C**). When 7.5 x 10^5^ CpG-proBs per recipient were injected on day+2 post-irradiation (DPI), they provided significant protection, as assessed by reduced diarrhea and less skin damage but no significant increase in survival compared to controls with GVHD (**Figure 1**). By contrast, the same number of non-activated pro-B cell progenitors freshly sorted from the bone marrow as c-kit^+^Sca-1^-^B220^+^CD24^hi^CD43^hi^ cells (**Supplementary Figure 1C**) and expanded in the same conditions had no such effect (**Figure 1**). The same number of CpG-proBs adoptively transferred on day+9 conserved a reduced but still significant protection against disease symptoms, which was lost when injected on day+23 (**Figure 1**).

CpG induced a strong upregulation of MHC class II, together with the co-stimulatory molecule CD80, as well as high CD40 expression on proB cell progenitors, thereby improving their capacity to interact with T-cells. There was no significant difference between CpG-proBs and their unstimulated counterpart, in terms of FasL expression, while PDL-1 was upregulated, compared with unstimulated controls, which did not display this molecule at significant levels (**Supplementary Figure 2A**). However, the difference between CpG-proBs and proBs became less pronounced after expansion on the OP-9 cell layer. Finally, FACS analysis of PMA+ionomycin-activated proBs and expanded CpG-proBs revealed no significant difference between their cytokine expression profiles (GM-CSF, TNF-α, IL-10 and IFN-γ) (**Supplementary Figure 2B**).

### CpG-proBs Migrate Into Peripheral Organs Where They Differentiate

**Figure 2:**
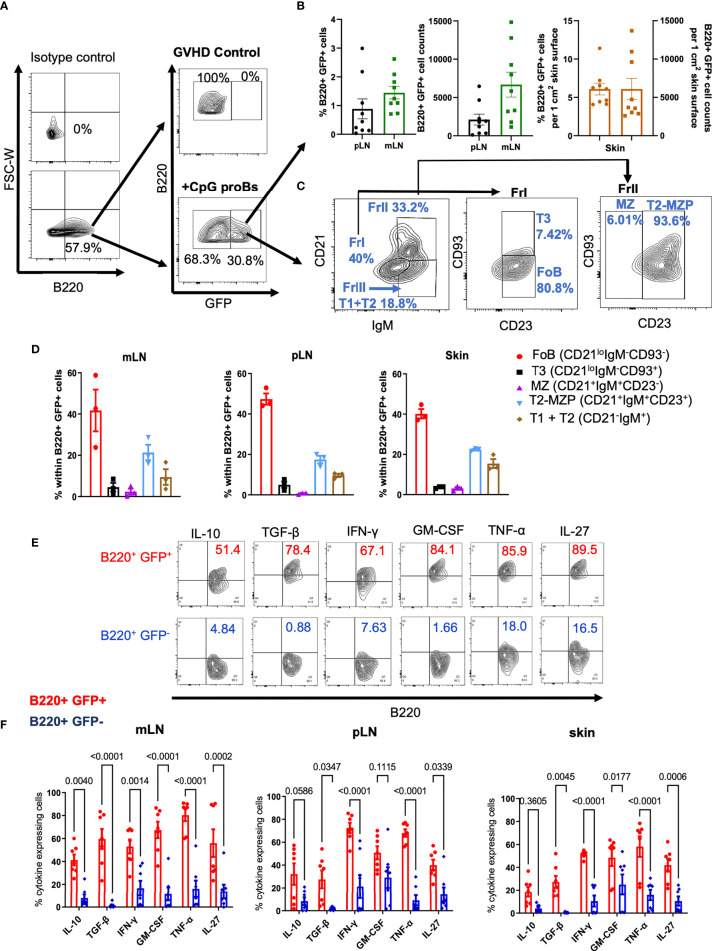
Migration, differentiation and cytokine expression of CpG-proBs in GVHD mice. CpG-proBs, isolated from the BM of actin-GFP-KI C57BL/6J donors, were adoptively transferred on day+2 post-irradiation. **(A)** Gating FACS procedure of B220^+^ GFP^+^ cells, shown on day+15 in mesenteric lymph nodes (mLN), in controls with GVHD and in CpG-proB recipients, isotype antibody controls being used to define positivity. **(B)** The migration of B220^+^GFP^+^ cells was traced and analyzed by FACS on day+15 in peripheral and mesenteric lymph nodes (pLN), mLN) and skin. Indicated are percentages of B220^+^GFP^+^ cells among all recovered cells. In the skin, percentages and counts of B220^+^GFP^+^ cells are indicated per 1 cm^2^ of skin surface. **(C, D)** Differentiation of CpG-proBs **(C)** and phenotype of the B220^+^GFP^+^ progeny assessed on day+15 in mLN. Isotype antibody controls were used to define positivity. The various B-cell subfractions were defined as FoB (CD21^lo^IgM^-^CD93^-^), T3 (CD21^lo^IgM^-^CD93^+^), MZ (CD21^+^IgM^+^CD23^-^), T2-MZP (CD21^+^IgM^+^CD23^+^) and T1+T2 (CD21^-^IgM^+^) cells. **(D)** CpG-proB differentiation on day+15 in mLN, pLN and skin. **(E, F)** Cytokine expression by the CpG-proB progeny on day+15. **(E)** FACS profiles of cytokine (IL-10, TGF-β, IFN-γ, GM-CSF, TNF-α and IL-27) expression by CpG-proB-derived B220^+^GFP^+^ and non-CpG-proB-derived B220^+^GFP^-^ cells in the mLN. **(F)** Percent cytokine expressing B220^+^GFP^+^ (red) and B220^+^GFP^-^ (blue) cells in mLN, pLN and skin. Statistical analysis was performed with two-way ANOVA with Bonferroni multiple comparisons. **(B, D, F)** Results are expressed as mean ± SEM of 3-9 mice per group.

We took advantage of CpG-proBs derived from actin-GFP-knock-in (KI) mice to track their migration in recipients. On day+15, B220^+^GFP^+^ cells, gated as in **Figure 2A**, represented 20-30% of all B cells analyzed and were detected exclusively in CpG-proB recipients, in mesenteric (mLN) and peripheral lymph nodes (pLN) as well as in the skin (**Figure 2B**). Using a gating strategy based on relative expression of IgM, CD21, CD23 and CD93 (20–22) in all tissues examined, approximately 40% B220^+^GFP^+^ cells displayed a CD21^low^CD23^+^CD93^-^IgM^+^ phenotype (**Figures 2C, D**), similar to follicular B (FoB) cells, previously identified as the major CpG-proB progeny in NOD mice (15).

### Cytokines Are Expressed in the Peripheral CpG-proB Progeny

Twenty to 80% B220^+^GFP^+^ cells expressed various cytokines, including IL-10, TGF-β, IFN-γ, GM-CSF, TNF-α and IL-27, compared with only 10-25% positive cells among the non-CpG-proB-derived B220^+^GFP^-^ population. These observations suggest that the CpG-proB cell progeny is highly activated, especially in mLN, in which B220^+^GFP^+^ cells expressing these cytokines, notably IL-10 and TGF-β, were more frequent than in their pLN and skin counterpart (**Figures 2E, F**).

### Characterization of Two Distinct Phases of Cytokine T-Cell Response in Mice With GVHD

**Figure 3:**
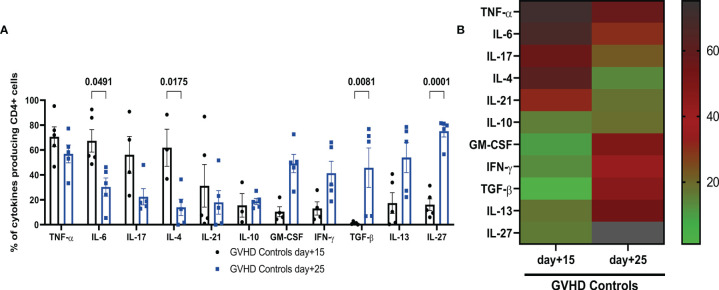
Characterization of two phases of cytokine expression by mLN CD4+ T-cell in controls with GVHD. **(A)** CD4^+^ T cells were stimulated by PMA + ionomycin in the presence of brefeldin and their intracellular cytokine expression was analyzed by FACS in mLN of GVHD controls at day+15 (black) and day+25 (blue). Data are expressed as means ± SEM of 5 mice per group. Statistical analysis was performed with two-way ANOVA with Bonferroni multiple comparisons. *p* values as indicated, n.s., non significant. **(B)** Heatmap representation of the mean of percentages of CD4^+^ T-cell expression of indicated cytokines in mLN of control mice with GVHD, at day+15 and day+25. Right: Colour scale of intensity of percentages.

We investigated whether the two diarrhea phases occurring in this GVHD model, the first one between day+2 and day+18, the second one starting at day+20, concomitant with the onset of skin damage, corresponded to an initial acute inflammatory cytokine storm followed by a chronic phase characterized by a more systemic autoimmune disease associated with the alteration of regulatory mechanisms due to the alloreactive conflict. To this aim, we investigated the CD4^+^ T-cell intracellular expression of cytokines by flow cytometry, in the mLN of control mice with GVHD at day+15 and day+25. Percentages of CD4^+^ cells (**Figure 3A**) expressing TNF-α, IL-6, IL-17 and to a lesser extent IL-21 were already high at day+15 while IL-6 and IL-4 were statistically reduced at day+25. IL-17 expression also tended to be reduced between day+15 and day+25 but without reaching statistical significance, while TNF-α remained highly expressed at day+25. Conversely, low levels of GM-CSF, IFN-γ, IL-13 and IL-27 expression with nearly no detectable expression of TGF-β by CD4^+^ T-cells were observed at day+15 while their expression was enhanced at day+25, with statistical significance for TGF-β and IL-27. IL-10 expression remained low and unmodified at day+15 and day+25. Such clear-cut shift in the cytokine expression pattern determining two distinct phases of the disease was even more conspicuous in a heatmap representation (**Figure 3B**). Therefore, the CD4^+^ T-cell response is significantly distinct at day+15 and day+25 in this GVHD model, with an initial inflammatory phase at day+15 followed by a pro-fibrotic cytokine production at day+25, characteristic of the chronic phase of GVHD. Consequently, it was interesting to evaluate the effect of the adoptive transfer of CpG-proBs at these two phases of the GVHD model, i.e. day+15 and day+25.

### CpG-proBs Modulate Cellular Distribution and Cytokine Expression in GVHD Recipients

**Figure 4:**
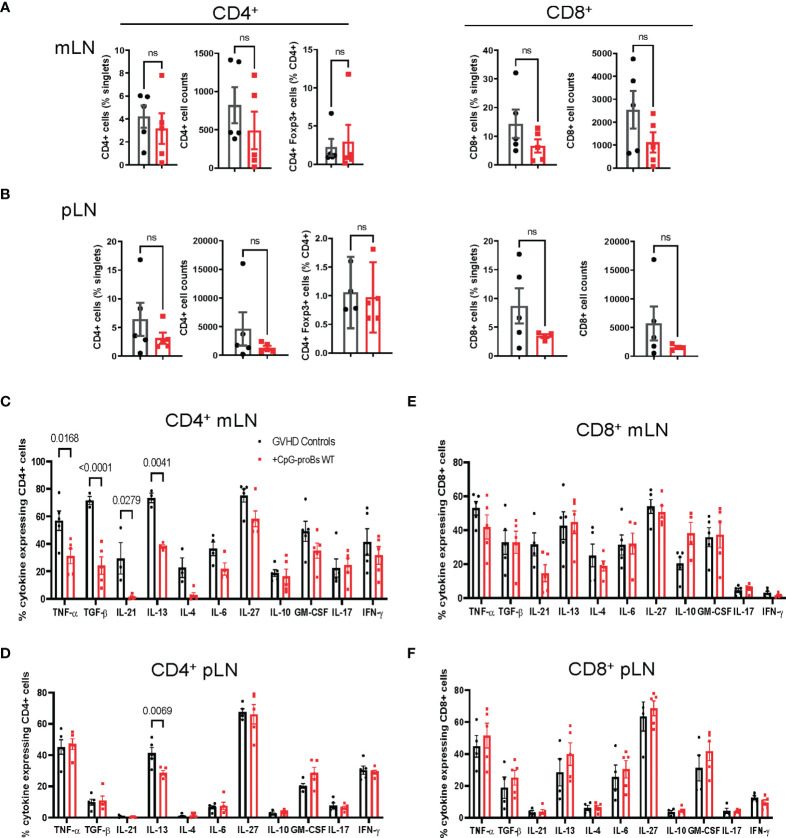
T-cell subset analysis in mLN and pLN of CpG-proB recipients and GVHD controls. **(A, B)** Quantification by FACS analysis on day+25 of CD4^+^, CD8^+^ (% and cell counts) and CD4^+^Foxp3^+^ (%) in mLN **(A)** and pLN **(B)** of GVHD controls (black) and CpG-proB recipients (red). **(C, D)** Cytokine expression by CD4^+^ T cells in mLN **(C)** and pLN **(D)** of GVHD controls (black) and CpG-proB recipients (red). Data are expressed as means ± SEM of 5 mice per group. Statistical analysis was performed with unpaired Students’*t*- test **(A, B)** and two-way ANOVA with Bonferroni multiple comparisons **(C–F)**. *p* values as indicated, n.s., non significant.

We analyzed the effect of adoptively transferred CpG-proBs on various recipient cell populations. On day+15, incidence and cell counts of CD4^+^ T cells or CD4^+^Foxp3^+^ Treg cells were neither significantly different from controls nor did the cytokine expression by CD4^+^ T-cells in mLN and pLN change (**Supplementary Figure 3A, B**). On day+25, once the chronic phase initiated, percentages of CD4^+^, CD4^+^Foxp3^+^ Treg and CD8^+^ T-cells as well as cell counts were not significantly modified (**Figures 4A, B**). However the proportion of CD4^+^ T cells generating cytokines, such as TNF-α, TGF-β, IL-21 and IL-13, which are critically involved in chronic GVHD (23), was significantly reduced in mLN from CpG-proB recipients (**Figure 4C**), while only IL-13-expressing CD4^+^ T cells were diminished in pLN (**Figure 4D**). No significant difference was noted for IL-10 expression in CD4^+^ cells (**Figures 4C, D** and **Supplementary Figure 3**), while it was slightly but non-significantly enhanced in mLN but not in pLN CD8^+^ T-cells (**Figures 4E, F**).

### Adoptive Transfer of CpG-proBs Increases the Tfr/Tfh Ratio

**Figure 5:**
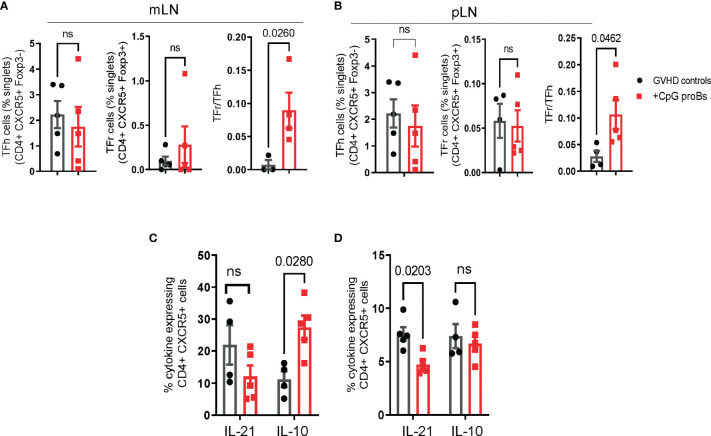
Follicular T-cell (Tf) analysis. **(A, B)** Percentages and counts of Tfh (CD4^+^CXCR5^+^Foxp3^-^) and Tfr (CD4^+^CXCR5^+^Foxp3^+^) cells as well as Tfr/Tfh ratios on day+25 in mLN **(A)** and pLN **(B)** of mice, either CpG-proB recipients (red) or GVHD controls (black), were established by FACS analysis. **(C, D)** Cytokine expression by CD4^+^CXCR5^+^ cells assessed by FACS analysis. Percent IL-21- and IL-10-expressing cells in mLN **(C)** and pLN **(D)** of GVHD controls (black) and CpG-proB recipients (red). Results are expressed as means ± SEM from 5 mice per group. Statistical analysis was performed with unpaired Students’*t*- test **(A, B)** and two-way ANOVA with Bonferroni multiple comparisons **(C, D)**. *p* values as indicated, ns= non significant.

T follicular helper (Tfh) cells, counterbalanced by T follicular regulatory (Tfr) cells, are known to play a key role in the CD4^+^ T-B cell interaction (24). In addition, Bregs have been reported for interacting with both Tfh and Tfr subsets (25, 26). This led us to examine how CpG-proBs and their progeny affected the balance between these two populations. Tfh evaluation on day+15 disclosed no difference between GVHD controls and CpG-proB recipients (**Supplementary Figure 3C**). Conversely, on day+25, the ratio between CD4^+^CXCR5^+^Foxp3^+^ follicular T regulatory cells (Tfr) and CD4^+^CXCR5^+^Foxp3^-^ follicular helper T (Tfh) cells was markedly increased in both mLNs (**Figure 5A**) and pLNs (**Figure 5B**) of CpG-proB recipients relative to their counterpart in control mice undergoing GVHD. Moreover, the percentage of Tfh cells expressing IL-10 was increased in mLN, while Tfh cells expressing IL-21 were diminished in pLN of mice having received CpG-proBs relative to untreated GVHD controls (**Figures 5C, D**). Finally, percentages of CD19^+^GL7^+^CD38^low^ GC B cells did not differ significantly in spleen and mLN (not shown). Altogether, these data show that CpG-proBs switch the follicular T-cell compartment towards regulation, by favoring the accumulation of Tfr over Tfh cells and by promoting their production of the immunoregulatory cytokine IL-10 over IL-21.

### The Protection Against GVHD by CpG-proBs Depends on IFN-γ Production

**Figure 6:**
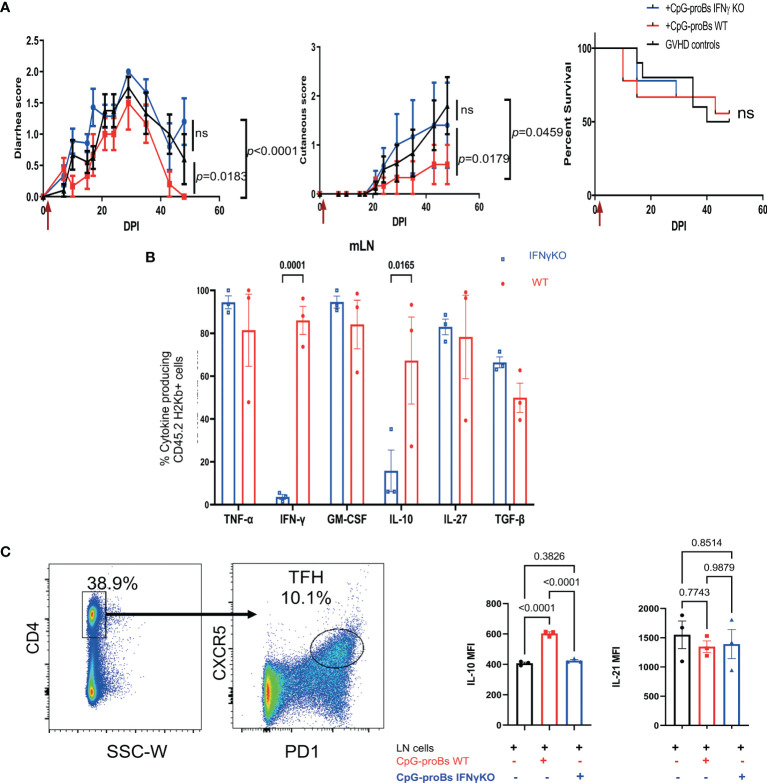
Role of IFN-γ in the protective properties of CpG-proBs against GVHD. CpG-proBs were prepared from either WT or IFN-γ deficient C57BL/6J donors and adoptively transferred (7.5 x 10^5^ cells/recipient) on day+2 post-irradiation (DPI). **(A)** Diarrhea, cutaneous scores and survival of GVHD controls (injected with PBS, black, n=10), WT CpG-proB recipients (red, n=9) and IFN-γ deficient CpG-proBs (blue, n=9). Statistical analysis was performed with two-way ANOVA with Bonferroni multiple comparisons for diarrhea and cutaneous scores. Results are expressed as means ± SEM. *P* values as indicated. ns= non significant. **(B)** CpG-proB progeny, derived from either WT (red) or IFN-γ deficient (blue) C57BL/6J CpG-proBs, was gated as CD45.2 H2Kb^+^ cells in mLN of GVHD Balb/c (H2Kd) recipients of CD45.1 TBCD-BM and splenocytes from CD45.1 C57BL/6J donors and their cytokine expression analyzed by FACS as in **Figure 2E** on day+15 after adoptive transfer. N=3 mice per group. **(C)** Lymph node cells from naive C57BL/6J mice were co-cultured at a 1:1 ratio with WT or IFN-γ KO CpG-proBs at 5 x 10^5^ cells/ml for 3 days in RMPI 1640 medium, 10% FCS, 1% antibiotics, 0.1% β-mercaptoethanol in the presence of anti-CD3 (200 ng/ml) and analyzed by FACS for IL-10 and IL-21 expression in gated CD4^+^CXCR5^+^ Tfh cells. One experiment out of two. Statistical analysis was performed with one-way ANOVA for **(B, C)** Results are expressed as means ± SEM. *p* values as indicated.

IFN-γ plays a key role in the protective effect of CpG-proBs in autoimmune T1D (15) and EAE (16). In GVHD mice, their migrated B220^+^GFP^+^ progeny expressed IFN-γ at similar levels, whatever the target tissue (**Figure 2F**), which prompted us to evaluate its role in the GVHD model. Using CpG-proBs isolated from IFN-γ-deficient mice, we found that graft recipients displayed exacerbated diarrhea and skin damage, compared with those having received WT CpG-proBs (**Figure 6A**). This finding proved the importance of IFN-γ in the protection against GVHD by CpG-proBs. The progeny of IFN-γ deficient CpG-proBs having migrated to the mLN did not express IFN-γ as expected, but also generated less IL-10, compared to its WT counterpart (**Figure 6B**). Moreover, co-culturing peripheral and mesenteric lymph node cells isolated from naive mice with CpG-proBs significantly enhanced IL-10 expression in gated CD4^+^CXCR5^+^PD1^+^ Tfh cells, only when the progenitors were competent IFN-γ producers (**Figure 6C** and **Supplementary Figure 5**).

### CpG-proBs Reduce Fibrosis and Regulate Gene Expression and Infiltrates in the Skin

**Figure 7:**
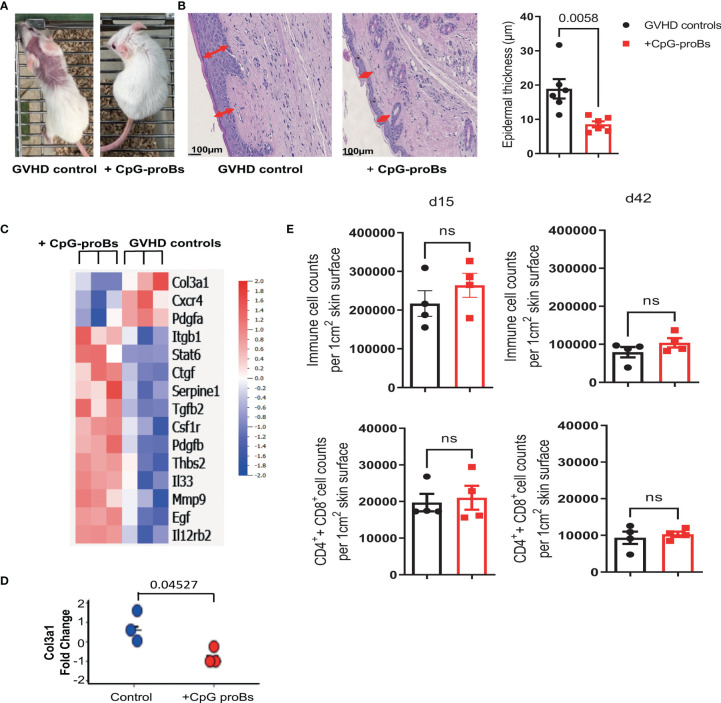
Analysis of skin histological modifications, gene expression and cellular infiltrate in GVHD controls versus CpG-proB recipients. **(A)** CpG-proB recipients were mostly protected from alopecia and skin damage induced by GVHD in Balb/c recipients. Picture at day+70 of one representative mouse per group. **(B)** H&E staining of representative skin sections at day+70 in GVHD controls versus CpG-proB recipients. Scale bar = 100 μm. Red arrows indicate the epidermal thickness. Forty measures were taken per skin section. Right: Histogram representation of epidermal thickness in GVHD controls (black) and CpG-proB recipients (red). Results are expressed as means ± SEM from 6 mice/group. *p* value as indicated. Analysis was performed with unpaired Student’s *t*-test. **(C)** Heatmap showing significant fold-change expression of genes as measured by qRT-PCR microarray in skin fragments (2 cm^2^) isolated at day+70 from GVHD controls (right) and CpG-proB recipients (left). N = 3 animals per group. Analysis was performed with Qlucore. Listed are genes showing ≥1.4 expression fold change with p ≤ 0.05, considered significant. Right: Color scale of positive and negative fold-change gene expression. **(D)** Change fold of Col3A1 mRNA expression measured by qRT-PCR in skin samples recovered at day+70 from n=3 animals per group. **(E)** Flow cytometry analysis on day+15 and day+42 of total immune cell infiltrates as well as T-cell (CD4^+^ and CD8^+^) infiltrates in skin samples of GVHD controls (black) and CpG-proB recipients (red). Results are expressed as means ± SEM from 4 mice per group. Statistical analysis performed with unpaired Student’s *t*-test, ns, non significant, *p* values as indicated.

**Figure 8:**
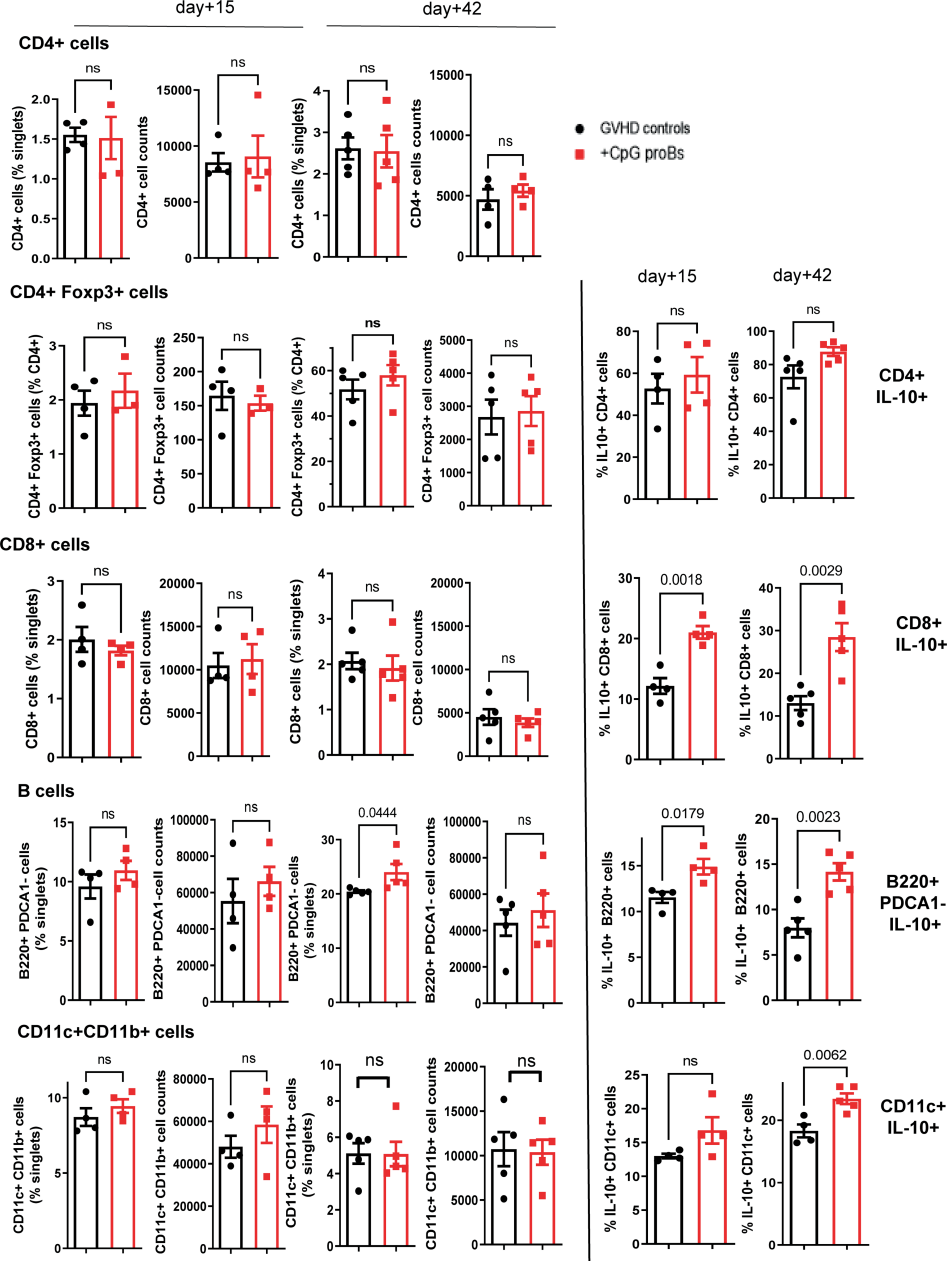
Flow cytometry analysis of skin infiltrates on day+15 and day+42 post-irradiation. CD4^+^, CD4^+^Foxp3^+^, CD8^+^, B220^+^PDCA-1^-^ B and CD11c^+^CD11b^+^ dendritic cell percentages and cell counts are shown in GVHD controls (black) and CpG-proB recipients (red). IL-10-expressing fraction of CD4^+^, CD8^+^, B220^+^ and CD11c^+^ cells on day+15 and day+42 in the skin of GVHD controls (black) and CpG**-**proB recipients (red). Results are expressed as means ± SEM for 5 mice per group. Statistical analysis was performed with unpaired Student’s *t*-test, ns, non significant. *p* values as indicated.

GVHD recipients of CpG-proBs developed less alopecia and skin damage (**Figure 7A** right) compared with GVHD controls at day+70 (**Figure 7A** left). Histological analysis of H&E-stained skin sections recovered on day+70 revealed 50% reduced epidermal thickness (**Figure 7B**), consistent with diminished skin fibrosis. In addition, hair follicles that are a target of GVHD (27) are preserved in the skin of CpG-proB recipients, correlating with the observed reduced alopecia (**Figure 7A**). qRT-PCR microarray expression profiles, established at day+70, of genes involved in fibrosis and cytokine production (**Figure 7C**) disclosed that *Col3a1* (**Figure 7D**) as well as of *Pdgfa*, a Col3a1 inducer implicated in fibrosis were downregulated in samples from CpG-proB recipients. The expression of Pdgfa, a known inducer of CXCR4 (28), which attracts fibrocytes to fibrotic tissues (29, 30) was likewise reduced in the skin of CpG-proB recipients. By contrast, thrombospondin-2 (*thsb2*, TSP-2), an anti-angiogenic matricellular protein that improves wound healing (31) was upregulated in CpG-proB recipients. The same applied to *MMP9*, which behaves like a collagenase (32) and can further regulate leukocyte infiltration into inflammatory tissues (33) by inactivating a number of chemoattractants. However, neither total immune cell nor T-cell infiltration was significantly different between GVHD controls and CpG-proB recipients on day+15 or day+42 (**Figure 7E**). The two-fold reduction in the total cell counts infiltrated at day+42 relative to day+15 (**Figure 7E**) observed in both control and CpG-proB-treated groups may reflect an initial transient wave of infiltration followed by a gradual inactivation of chemoattractants ligands or receptors occurring in the second phase of the model with profibrotic events taking over, thus contributing to enhance the cutaneous score observed in **Figure 1**. The enhanced *IL12rb* expression suggested a proTh1 effect of CpG-proBs on skin infiltrates, possibly controlling the deleterious Th2-driven fibrotic process. This conclusion was in keeping with the observed decrease in IL-13 expression by CD4^+^ T-cells in the lymph nodes. Increased Stat6 expression in CpG-proB recipients (**Figure 7C**) was intriguing, knowing that this signal transducer can mediate skin fibrosis (34). However, this upregulation might result from increased expression of IL-33, which occurs upstream of IL-13 (35). Of note, IL-33 can substitute for IL-2 as an inducer of tissue ST2^+^ Treg expansion (36). Although the proportions of CD4^+^Foxp3^+^ Tregs and CD4^+^IL-10^+^ Tr1 cells were not significantly increased in skin infiltrates, as measured by FACS analysis (**Figure 8**), IL-10-expressing CD8^+^ T cells, reported for their ST-2 expression and responsiveness to IL-33 (37), markedly accumulated in the skin of CpG-proB recipients, both on day+15 and day+42, while total CD8^+^ T-cell counts and percentages remained unchanged (**Figure 8**).

The proportion of IL-10 producers increased also among the B220^+^PDCA-1^-^ B subset as early as day+15, while on day+42, both B220^+^PDCA-1^-^ B cells and CD11c^+^CD11b^+^ dendritic cells expressing IL-10 accumulated (**Figure 8**). During GVHD, macrophages stimulated by Fc immunoglobulin fragments contribute to fibrosis by releasing TGF-β. *Csf1r* was enhanced in the microarray analysis of skin samples from CpG-proB recipients. However, FACS analysis of the skin cell infiltrate revealed that cell counts, percentages as well as IL-10 production by F4/80^+^CD11b^+^ macrophages remained unchanged on day+42 (**Supplementary Figure 4**). Moreover, microarray analysis detected no significant difference between *Arg* and *iNOS* expression. In mice, CSF1R is expressed by monocytes/macrophages, but also by conventional and plasmacytoid dendritic cells. However, the observed incremental increase in conventional (**Figure 8**) and plasmacytoid dendritic cell percentages and IL-10 expression (**Supplementary Figure 6**) did not reach statistical significance. A late accumulation of csf1r^+^ cells in the skin analyzed on day+70, compared to the flow cytometry analysis performed on day+42, cannot be excluded.

Collectively, the analysis of skin samples and infiltrates revealed the histological effects of CpG-proBs resulting in reduced skin damage, including fibrosis, epidermal thickness and collagen accumulation. These findings correlated with immune tolerance evidenced by enhanced infiltration by IL-10-expressing DCs, CD8^+^ T cells and B cells. The two latter populations were first to accumulate in the skin.

## Discussion

Herein, we evaluated whether adoptive transfer of CpG-activated B-cell progenitors exerted immunomodulatory effects in a model of GVHD that displays sequentially an acute and a chronic sclerodermatous phase (18). This study was initiated by recent evidence for Breg deficiencies and impaired functions in patients suffering from this disease (3, 7), together with the observation that circulating hematogones and protection against GVHD (8–14) were correlated. These findings warranted further exploration of the regulatory functions of B-cell progenitors in the allogeneic model of GVHD, expanding our previous studies in experimental models of autoimmune diseases, such as T1D (15) and EAE (16).

A single injection of as few as 7.5 x 10^5^ CpG-proBs was sufficient to protect against GVHD, by reducing diarrhea and skin fibrosis within a therapeutic window extending from day+2 up to day+9. The effect vanished when these cells were injected on day+23, indicating that they must intervene during the onset of disease to prevent its chronic phase. The easy access to the B-cell progenitors within the BM at the time of engraftment should facilitate their potential use as an addition to the HSC graft, as they provide long-lasting protection against diarrhea and skin fibrosis in GVHD. Protection required around 10-fold higher CpG-proB cell numbers than those needed in the case of organ-specific autoimmune disorders, presumably reflecting the necessity to migrate into the multiple tissues implicated in the allogeneic immune response.

Indeed, CpG-proB progeny was detected in the target sites of GVHD, including mLN, pLN and skin, as early as day+15, mainly differentiated into Fo B cells, as previously observed in the T1D model of NOD mice (15). Compared to non-CpG-proB-derived B cells in the same locations, the differentiated CpG-proBs were highly activated, as assessed by a 2-8 fold higher proportion of cells expressing cytokines, such as IFN-γ, GM-CSF, TNF-α, as well as IL-10, TGF-β and IL-27. Among these, IFN-γ production by CpG-proBs and their progeny proved to be critical for alleviating GVHD symptoms, particularly skin fibrosis, as previously shown in experimental models of autoimmune diseases, such as T1D (15) and EAE (16).

While CpG-proBs had to be adoptively transferred during the initial phase of GVHD for protection, their effect on the T-cell cytokine profile was observed mostly on day+25, when the expression of CD4^+^ T-cell-derived cytokines involved in the inflammatory, humoral and fibrotic features of the chronic phase of GVHD, such as TNF-α, IL-21, TGF-β and IL-13, was significantly reduced in mLNs and pLNs of CpG-proB recipients compared to controls with GVHD. However, no effect was observed on GM-CSF, IL-17 and IFN-γ expression by CD4^+^ T-cells in the mLN. The unmodified T-cell expression of these highly inflammatory cytokines in CpG-proB recipients may account for the lack of effect of the progenitors on mice survival. It remains to be evaluated whether performing a second progenitor cell transfer during the chronic phase might improve the mice survival. Alternatively, these observations suggest that CpG-proBs infusion should be associated with a supplementary strategy targeting anti-inflammatory cytokines beyond TNF-α, to be fully effective against GVHD. Conversely, as early as day+15, IL-10-expressing B cells and CD8^+^ T-cells accumulated in the skin of CpG-proB recipients, suggesting an early major contribution of these cells to the protective effect induced by CpG-proBs particularly in skin. In both murine (38, 39) and human (40, 41) GVHDs, IL-10-expressing CD8^+^ T cells have been reported for their regulatory effects, in particular for reducing collagen deposition in the skin of recipient mice (38). In the same line of evidence, we found IL-10-expressing dendritic cells accumulating on day+42 in the skin of CpG-proB recipients.

Fo B cells participate in germinal center (GR) responses generating long-lived plasma cells and memory B cells. The Tfh/Tfr balance plays a major role in GVHD, since Tfr cells can inhibit the interplay between Tfh and GC B cells (25, 26, 42–44). Bregs have been shown to take part in the crosstalk between these subsets (25, 26, 42). We found that the CpG-proB progeny belonged mostly to the Fo B phenotype and increased the Tfr/Tfh ratio. IFN-γ was essential for the capacity of the CpG-proB progeny to express IL-10 and enhance IL-10 expression by Tfh cells. We have previously reported that CpG-proB-derived IFN-γ induced eomesodermin in co-cultured CD4^+^ T-cells (15). In turn, EOMES drives IL-10 expression, as shown in Tr1 cells that are protective against GVHD (45). Whether a similar mechanism takes place in Tfh cells remains to be assessed. Notably, an IL-10 expressing Tfh cell population with suppressive function was identified in chronic viral infection (46) as well as in inflammation associated with aging (47). Thus, CpG-proBs exert a profound influence on major participants of the CD4^+^ T-B cell interaction that may limit the humoral response and IFN-γ production by CpG-proBs is required in both autoimmune and allogeneic settings.

IL-33 expression was enhanced in the microarray qRT-PCR study of skin tissue samples performed at day+70. Even though it has been reported that IL-33, released by epithelial and endothelial cells, induces cutaneous fibrosis, promoting the recruitment of BM-derived eosinophils as well as CD3^+^ and F4/80^+^ cell infiltration (48), we observed no accumulation of these cell types. Alternatively, IL-33 has also been described for its capacity to expand and stabilize ST2-expressing Tregs in tissues, thereby favoring tissue remodeling (36, 49). Treg frequency is inversely correlated with GVHD in patients (2, 50). Although we detected no accumulation of Foxp3^+^ Tregs in CpG-proB recipients compared to GVHD controls, IL-10^+^CD8^+^ Tregs were more frequent early in the skin of CpG-proB recipients. These IL-10^+^CD8^+^ Tregs, which reportedly express ST2 (37), may play a key role in GVHD recovery. Interestingly, IL-10^+^CD8^+^ Tregs were shown to contribute to the GVL effect in allogeneic HSCT (51). In addition, Bregs were reported not to compromise GVL effects while protecting against acute GVHD (52). Altogether these observations suggest that it is likely that CpG-proBs, like mature Bregs, may not impair the GVL effect of HSCT.

cGVHD is characterized by the presence of hyperactivated B cells (53). Conversely, circulating Bregs are less frequent in cGVHD patients and less likely to produce IL-10 than those from healthy donors (3). In a murine sclerodermatous cGVHD model, reconstitution of donor-derived B10 cells participated in alleviating the disease (54). Interestingly, IFN-γ competence conditioned both IL-10 expression by the CpG-proB progeny and its protective effect against disease. Most Breg subsets reported so far for protective effects in cGVHD were mature B cells. Even cord blood B cells displaying regulatory functions against cGVHD belonged to naive and transitional B-cell subsets (6). Although an intriguing inverse correlation between BM and circulating B-cell progenitor frequencies and GVHD severity has been reported, evidence for a regulatory function of B-cell progenitors in GVHD has been lacking so far. Our findings acquired in a murine experimental model support the notion that innate activation with CpG confers tolerogenic properties to B-cell progenitors. Their immunomodulatory effect targets more specifically the chronic phase of the disease that exhibits autoimmune inflammatory features, whereas no effect was observed at the early phase. However, the lack of reduction of major pro-inflammatory cytokines such as GM-CSF, IL-17 and IFN-γ may preclude an improved survival in CpG-proB recipients. Evaluation of CpG-proBs in a more specific cGVHD model would also be interesting to perform.

The fact that the observed regulatory properties of CpG-proBs remain stable in highly inflammatory settings sheds a new light on Breg ontogeny (55). In depth examination of epigenetic and metabolic changes occurring in these B-cell populations may provide further insights into their tolerogenic imprinting.

## Conclusion

**Figure 9:**
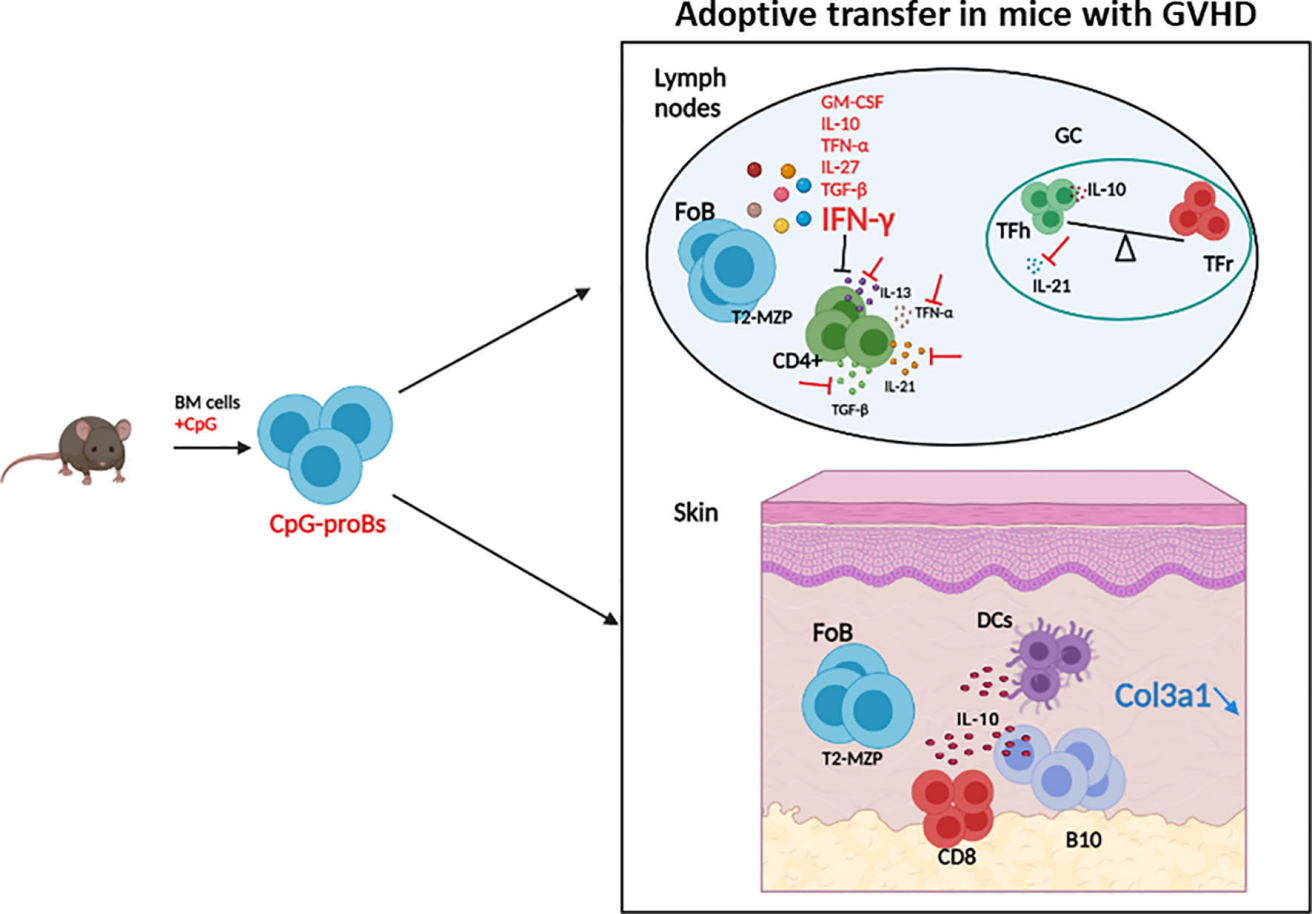
Graphical summary of the protective effects of CpG-proBs against GVHD. Adoptive transfer of CpG-proBs at the early phase of GVHD alleviated disease symptoms, in particular skin fibrosis. Following their migration into lymph nodes and skin, these progenitors produced many cytokines but depended on IFN-γ production for their protective effect. CpG-proB transfer reduced the CD4^+^ T-cell production of profibrotic cytokines TGF-β, IL-21 and IL-13 and enhanced the Tfr/Tfh T-cell ratio in lymph nodes. They also promoted the accumulation of IL-10-producing CD8^+^ T-cells, B-cells and dendritic cells in the skin. Figure created using BioRender.com.

In this study we provided evidence that adoptive transfer of CpG-proBs at the early phase of GVHD alleviated disease symptoms, in particular skin fibrosis. Following their migration into lymph nodes and skin, these progenitors depended on IFN-γ production for their protective effect, as previously shown in experimental models of autoimmune diseases. CpG-proB transfer reduced the CD4^+^ T-cell production of profibrotic cytokines, including TGF-β, IL-21 and IL-13 and enhanced the Tfr/Tfh T-cell ratio in lymph nodes. They also promoted the accumulation of IL-10-producing B-cells, dendritic cells and CD8^+^ T-cells in the skin (**Figure 9**). However, they did not improve survival, presumably by failing to reduce a set of inflammatory cytokines. Taken together, our data support a potential benefit of CpG-proBs against GVHD that should be completed by an additional anti-inflammatory strategy. The data further suggest that circulating B-cell progenitors observed to correlate with reduced GVHD severity in patients may play an immunomodulatory role.

## Data Availability Statement

The datasets presented in this study can be found in online repositories. The names of the repository/repositories and accession number(s) can be found below: https://www.ncbi.nlm.nih.gov/geo/, GSE182025.

## Ethics Statement

The animal study was reviewed and approved by Université Paris Cité Ethical Committee for Animal Experimentation and the French Ministry of Research and Higher Education, #21669-201807061804480v5.

## Author Contributions

VAA, PG, ET, and FZ performed experiments, analyzed data and prepared figures. SK provided expertise in the model, analyzed and discussed data. VAA, SK, and FZ wrote the manuscript. All authors contributed to the article and approved the submitted version.

## Funding

This work was supported by core funding from CNRS and INSERM. It was also funded by grants to FZ from Agence Nationale de la Recherche (ANR-17-CE17-0008), from Fondation pour la Recherche contre le Cancer (ARC), and from The Secular Society (TSS). VA was supported by a doctoral fellowship from TSS.

## Conflict of Interest

The authors declare that the research was conducted in the absence of any commercial or financial relationships that could be construed as a potential conflict of interest.

## Publisher’s Note

All claims expressed in this article are solely those of the authors and do not necessarily represent those of their affiliated organizations, or those of the publisher, the editors and the reviewers. Any product that may be evaluated in this article, or claim that may be made by its manufacturer, is not guaranteed or endorsed by the publisher.

## Abbreviations

aGVHD, acute GVHD; BM, bone marrow; Bregs, regulatory B cells; cGVHD, chronic GVHD; CpG-proBs, CpG-activated proB cell progenitors; EAE, experimental autoimmune encephalomyelitis; Fo B, follicular B cells; GC, germinal center; HSC, hematopoietic stem cells; HSCT, hematopoietic stem cell transplantation; mLN, mesenteric lymph nodes; pLN, peripheral lymph nodes; T1D, Type 1 diabetes; TBCD-BM, T-and B-cell depleted bone marrow; TFh, follicular helper T cells; TFr, follicular regulatory T cells; TGF-β, Transforming growth factor-beta; Treg, regulatory T cells; SPF, specific pathogen free.
