# Supplementary material for: CpG-Activated Regulatory B-Cell Progenitors Alleviate Murine Graft-Versus-Host-Disease
Source: Front Immunol. 2022 Apr 11;13:790564. doi: 10.3389/fimmu.2022.790564 (PMC9035844; doi:10.3389/fimmu.2022.790564)
Supplement: Supplementary file 1 [file DataSheet_1.pdf]

Agbogan V.A et al, Supplementary figure 1

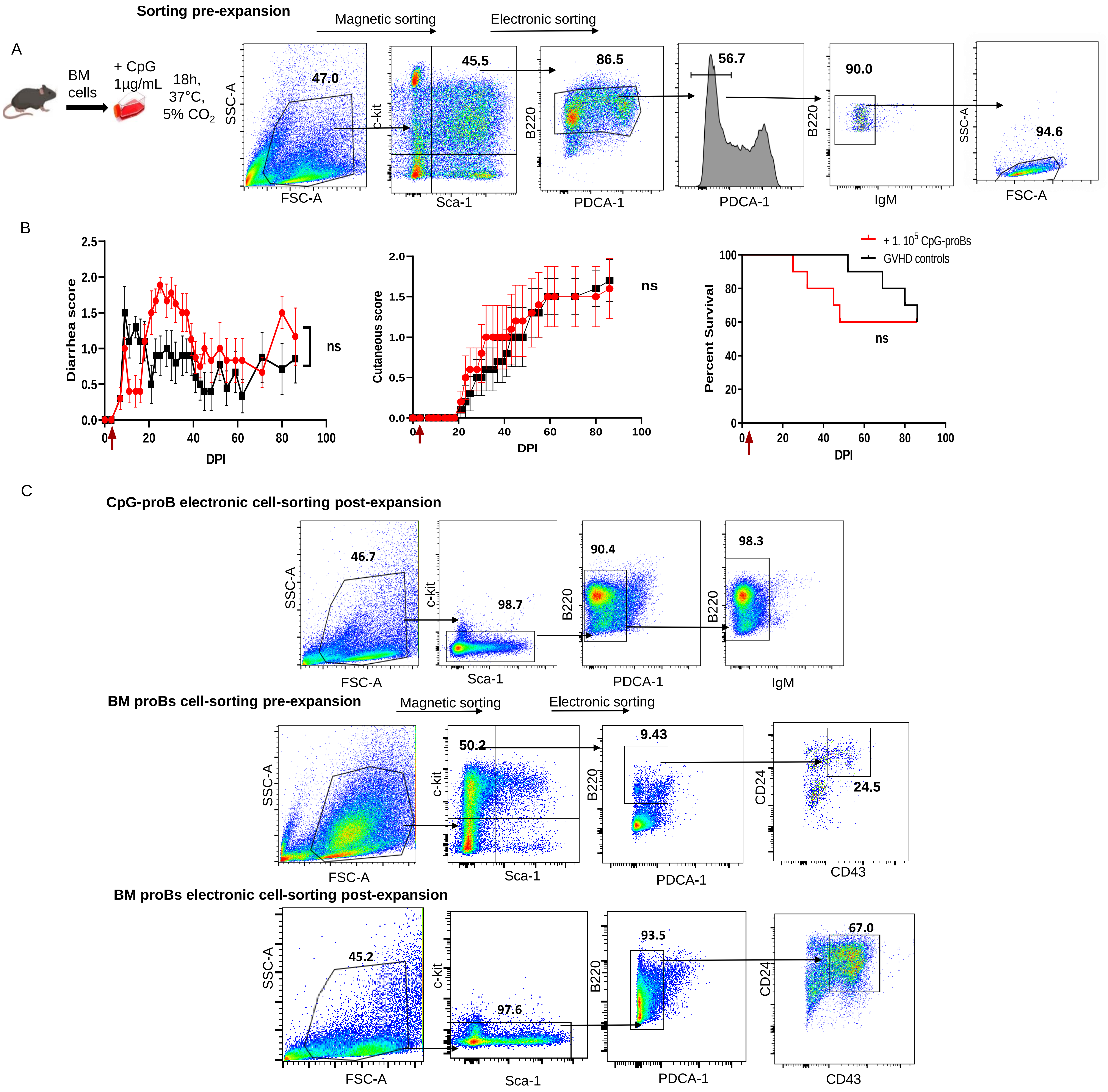

Supplementary Figure 1. (A) Cell culture and cell-sorting procedure of CpG-proBs. **(B)** Evaluation of CpG-proBs adoptively transferred at day+2 at 1 x 10^5 cells per recipients in mice with GVHD: left, diarrhea score; center, cutaneous score, both ns, by two-way ANOVA with Bonferroni post-test; right, survival curve, ns, by Kaplan Meier estimates, N=10 mice per group. **(C)** Upper panel: electronic cell-sorting of CpG-proBs post-expansion over OP-9 cells; middle panel: Cell-sorting of proBs isolated from fresh BM; lower panel: Electronic cell-sorting of BM proBs after expansion over OP-9 cells.

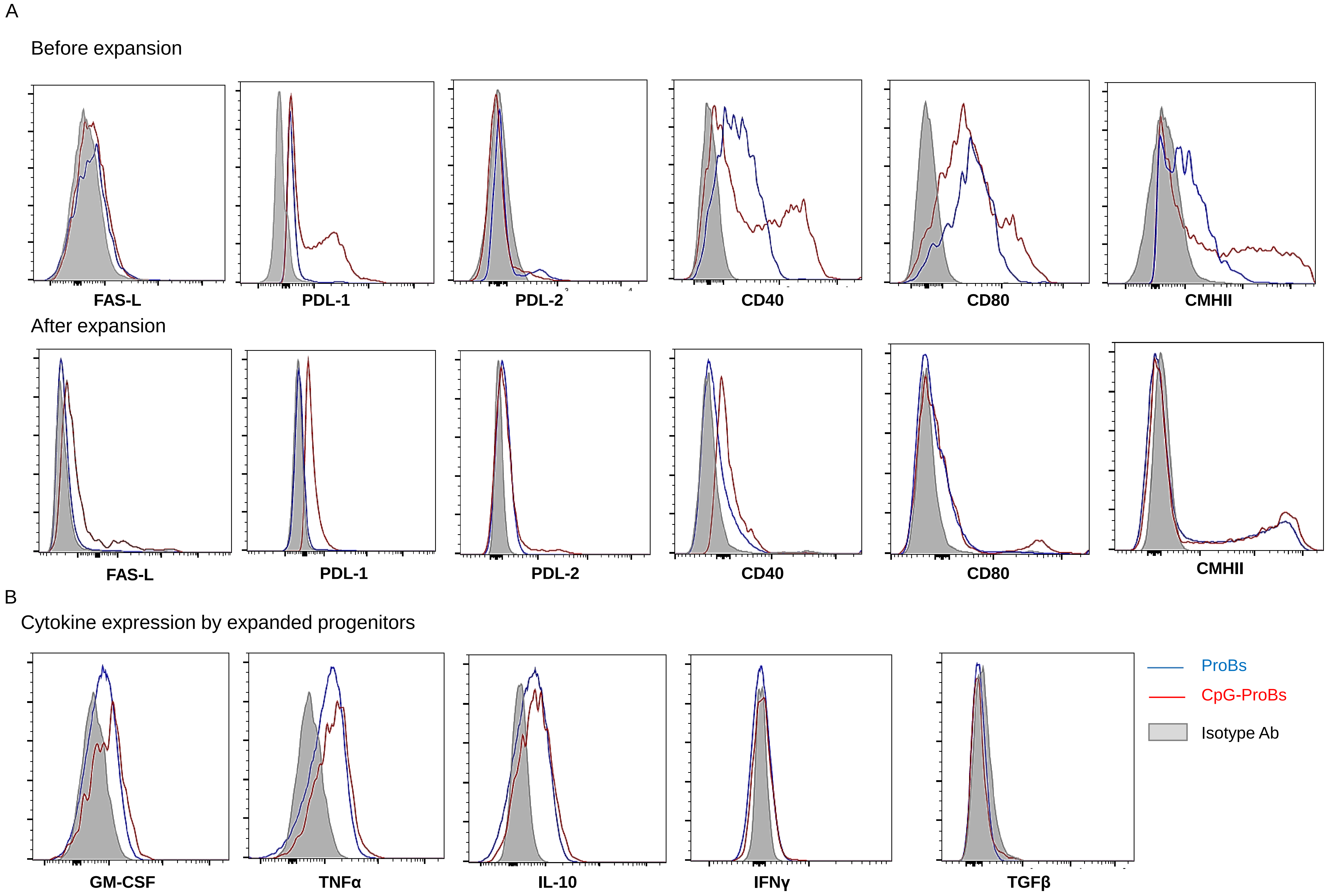

Supplementary Figure 2. (A) Cell-sorted CpG-proBs (red) and pro-Bs (blue) were compared for their expression of death and anergy inducing molecules as well as co-stimulatory markers before and after expansion over OP-9 stromal cells. **(B)** CpG-proBs (red) and ProBs (blue) were compared for their intracellular cytokine expression after activation for 4h with PMA + ionomycin. **(A, B)** Positivity was determined by comparison with isotype antibody staining (grey).

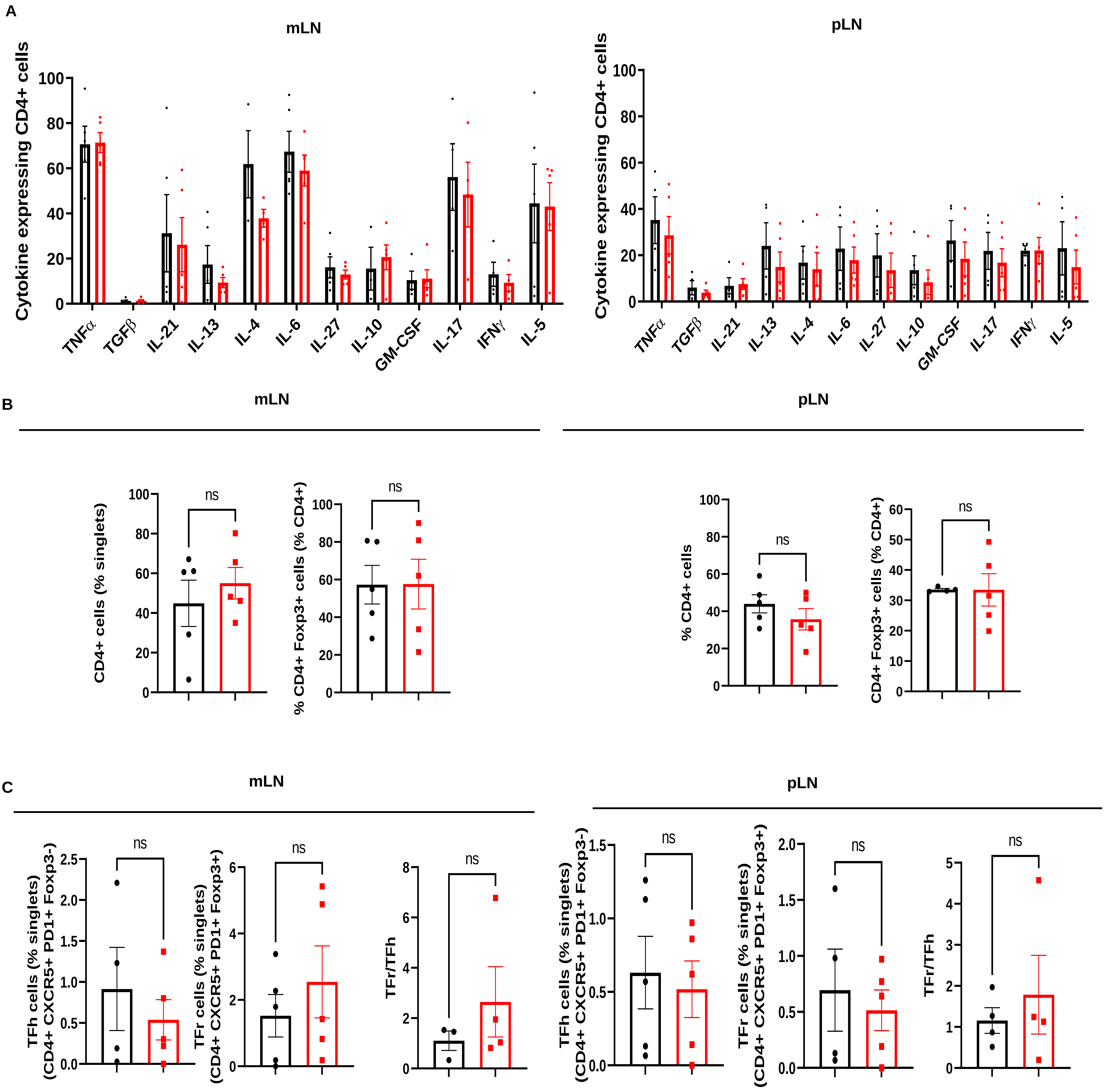

Supplementary Figure 3. GVHD control mice (black) and CpG-proB recipients (red) were compared at day+15 for **(A)** Intracellular cytokine expression by $CD4^+$ T-cells in the mLN and pLN. **(B)** Proportions of $CD4^+$ T-cells and $CD4^+Foxp3^+$ T-cells in the mLN and pLN. **(C)** Proportions of follicular T helper cells (TFh) ($CD4^+PD1^+CXCR5^+Foxp3^-$), follicular T regulatory cells ($CD4^+PD1^+CXCR5^+Foxp3^+$) and TFr/TFh ratio measured by flow cytometry in the mLN and pLN. ns= non significant, statistical analysis by (A) two-way ANOVA and (B, C) unpaired Students' *t*-test.

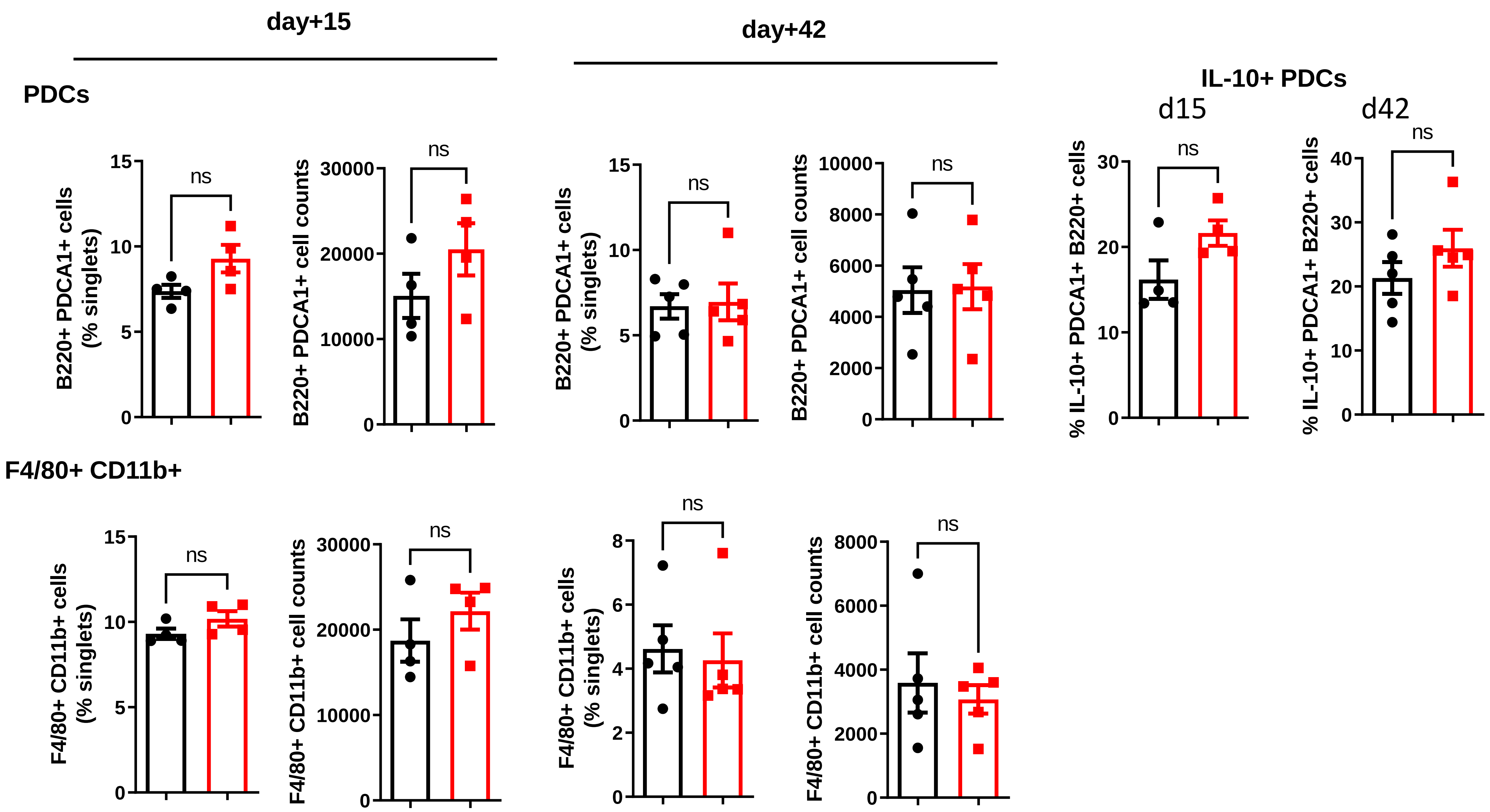

Supplementary Figure 4. Cells recovered from the skin of GVHD controls (black) and CpG-proB recipients (red) were analyzed by flow cytometry for their content in plasmacytoid dendritic cells (PDCS: B220^{+}PDCA-1^{+} cells) and macrophages (F4/80^{+}CD11b^{+} cells). Data represent mean ± SEM of their percentages and cell counts and of their IL-10 expression at day+15 and day+42 post-irradiation, as indicated. Ns, non significant, analyzed by unpaired Students' *t*-test.

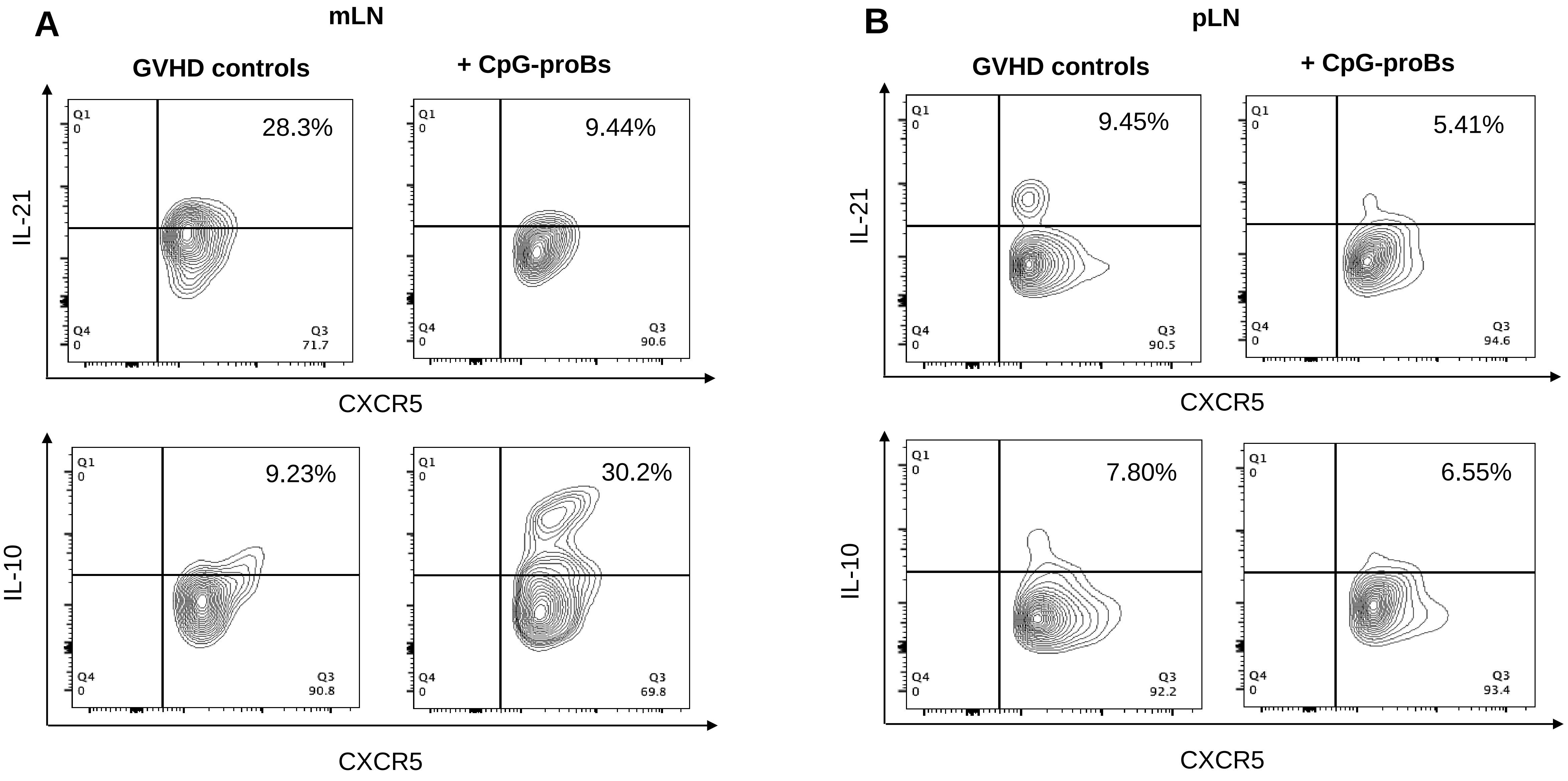

Supplementary Figure 5. (A-B) Representative FACS profiles of IL-21 and IL-10 expression by TFh cells ($CD4^{+}$ $CXCR5^{+}$) in the mLN **(A)** and pLN **(B)**

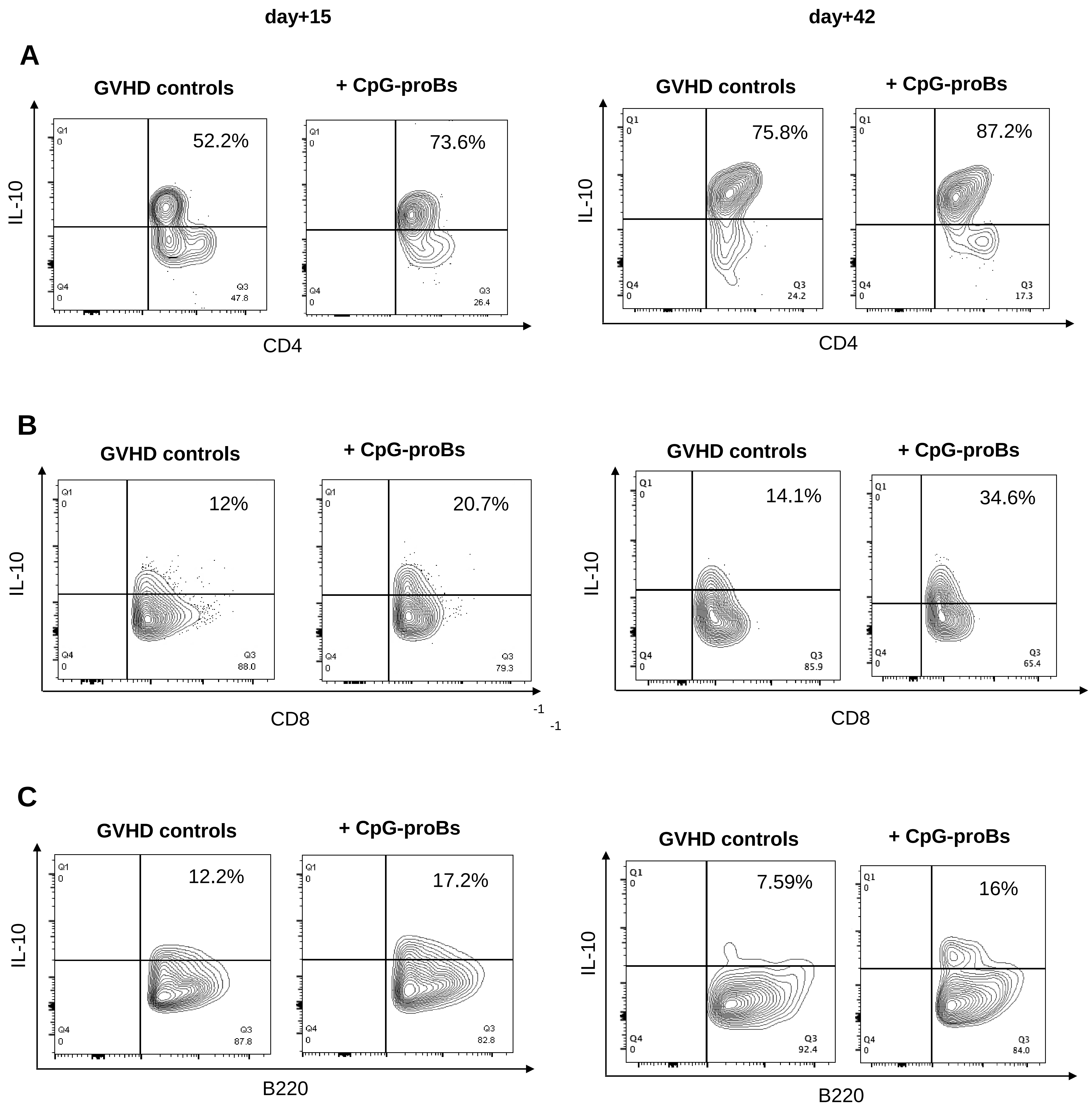

Supplementary Figure 6. (A-C) Representative FACS profiles of IL-10 expression at day+15 (left) and day+42 (right) by $CD4^+$ cells **(A)**, $CD8^+$ cells **(B)** and $B220^+$ $PDCA\text{-}1^-$ cells **(C)** from skin infiltrates.
